# Chyle Leak After Pancreatoduodenectomy: Clinical Impact and Risk Factors in a Nationwide Analysis

**DOI:** 10.1097/SLA.0000000000005449

**Published:** 2022-07-04

**Authors:** Simone Augustinus, Anouk E.J. Latenstein, Bert A. Bonsing, Olivier R. Busch, Bas Groot Koerkamp, Ignace H.J.T. de Hingh, Vincent E. de Meijer, I. Quintus Molenaar, Hjalmar C. van Santvoort, Judith de Vos-Geelen, Casper H. van Eijck, Marc G. Besselink

**Affiliations:** * Department of Surgery, Amsterdam UMC, Location University of Amsterdam, Amsterdam, The Netherlands; † Cancer Center Amsterdam, Amsterdam, The Netherlands; ‡ Department of Surgery, Leiden University Medical Center, Leiden, The Netherlands; ₧ Department of Surgery, Erasmus MC Cancer Institute, Erasmus University Rotterdam, Rotterdam, The Netherlands; ∥ Department of Surgery, Catherina Hospital, Eindhoven, The Netherlands; ¶ Department of Surgery, University Medical Center Groningen and University of Groningen, Groningen, The Netherlands; # Department of Surgery, Regional Academic Cancer Center Utrecht, University Medical Center Utrecht & St Antonius Hospital Nieuwegein, Utrecht, The Netherlands; ** Department of Internal Medicine, GROW—School for Oncology and Developmental Biology, Maastricht UMC+, Maastricht, The Netherlands

**Keywords:** pancreatic surgery, complications, chyle leak, outcomes

## Abstract

The aim of this study was to assess the clinical impact and risk factors of chyle leak (CL).

**Background:**

In 2017, the International Study Group for Pancreatic Surgery (ISGPS) published the consensus definition of CL. Multicenter series validating this definition are lacking and previous studies investigating risk factors have used different definitions and showed heterogeneous results.

**Methods:**

This observational cohort study included all consecutive patients after pancreatoduodenectomy in all 19 centers in the mandatory nationwide Dutch Pancreatic Cancer Audit (2017–2019). The primary endpoint was CL (ISGPS grade B/C). Multivariable logistic regression analyses were performed.

**Results:**

Overall, 2159 patients after pancreatoduodenectomy were included. The rate of CL was 7.0% (n=152), including 6.9% (n=150) grade B and 0.1% (n=2) grade C. CL was independently associated with a prolonged hospital stay [odds ratio (OR)=2.84, 95% confidence interval (CI): 1.85–4.36, *P*<0.001] but not with mortality (OR=0.3, 95% CI: 0.0–2.3, *P*=0.244). In multivariable analyses, independent predictors for CL were vascular resection (OR=2.1, 95% CI: 1.4–3.2, *P*<0.001) and open surgery (OR=3.5, 95% CI: 1.7–7.2, *P*=0.001). The number of resected lymph nodes and aortocaval lymph node sampling were not identified as predictors in multivariable analysis.

**Conclusions:**

In this nationwide analysis, the rate of ISGPS grade B/C CL after pancreatoduodenectomy was 7.0%. Although CL is associated with a prolonged hospital stay, the clinical impact is relatively minor in the vast majority (>98%) of patients. Vascular resection and open surgery are predictors of CL.

Chyle leak (CL) is a well-known complication after abdominal surgery, caused by disruption of abdominal lymphatics.^1^ In pancreatic surgery, CL is mostly caused by a direct lesion of the main abdominal lymphatic vessels or the cisterna chyli, which are located at the level of the pancreatic head and neck.^2^ It has been suggested that the incidence of CL after pancreatic surgery may increase because of the increasing numbers of extended resections.^3^

The rate of CL for pancreatic surgery in current literature ranges from 0.6% to 16.3%.^4^–^10^ Unfortunately, these studies used different definitions for CL. In 2017, the International Study Group of Pancreatic Surgery (ISGPS) published the consensus definition and classification of CL after pancreatic surgery, in which CL is defined as the output of milky-colored fluid from a drain, drain site, or wound on or after postoperative day 3 with a triglyceride content >110 mg/dL (>1.2 mmol/L). The classification divides CL into 3 grades of severity wherein grade B and C have clinical consequences, such as nasoenteral nutrition and or total parental nutrition (TPN) with medium-chain triglycerides (MCT), radiological interventions, and maintenance of surgical drains or drug treatment (such as somatostatin analogs). Grade C includes other invasive in-hospital treatments such as admission to the intensive care unit, and/or mortality.^3^

The ISGPS definition of CL has been validated in a monocenter retrospective study, which found a 3.5% rate of grade B/C CL but could not identify risk factors.^11^ In addition, a prospective monocenter study including 168 patients with serous drainage (appearance of clear fluid with no evidence for CL) and 60 patients with ISGPS CL, did not identify risk factors.^12^ Retrospective studies, using older definitions, identified multiple risk factors, including age, body mass index (BMI), lymph node status, resection margin, operative time, pathology results, para-aortic lymph node sampling or extend of lymphadenectomy, early enteral feeding, portal or mesenteric thrombosis, vascular resection, preexisting diabetes, and concomitant postoperative pancreatic fistula (POPF).^4^,^8^,^10^ Moreover, in these studies CL was associated with a prolonged hospital stay.^4^,^11^–^13^

Nationwide multicenter studies providing real-world data to validate the new ISGPS definition and classification of CL, including risk factors and postoperative outcomes associated with CL are lacking. Therefore, the aim of this study was to assess the incidence, clinical impact (ie, on length of stay or mortality), and risk factors of CL after pancreatoduodenectomy (PD).

## METHODS

### Study Design

This was a nationwide, observational cohort study of prospectively collected data from the Dutch Pancreatic Cancer Audit (DPCA).^14^ The DPCA is a mandatory audit for all centers that perform pancreatic surgery in The Netherlands, which collaborate in the Dutch Pancreatic Cancer Group (DPCG). The DPCA includes all patients who are scheduled for elective pancreatic surgery because of a (suspected) pancreatic or periampullary tumor, or pancreatic cysts. Excluded are pancreatic resections for chronic pancreatitis and pancreatic resections for tumors outside the pancreas.^15^ In 2017, 19 centers performed pancreatic surgery and during the study period one center stopped and 2 centers merged, resulting in 17 centers in 2019. In this analysis all patients after PD between 2017 and 2019 in The Netherlands were included. Patients undergoing PD before 2017 were not included, because the ISGPS classification of CL had not been implemented within the DPCA at that time. Patients with missing data on CL were excluded from analysis (1.2%). The study was reported in accordance with the STROBE guidelines.^16^

### Data Collection and Definitions

Data collected included patient characteristics (ie, age, sex, BMI, preoperative resectability status, American Society Anesthesiologists score), treatment characteristics (ie, neoadjuvant therapy, surgical approach, hospital PD volume, vascular resection, additional organ resection, aortocaval lymph node resection), tumor characteristics (ie, site of origin, postoperative malignant diagnosis, resection margin, lymph node status, number of lymph nodes resected), and postoperative outcomes (ie, CL, POPF, bile leak, postpancreatectomy hemorrhage, delayed gastric emptying, postoperative complications, pneumonia, wound infection, length of stay, in-hospital mortality). Preoperative resectability was defined according to the DPCG criteria.^17^ Venous resections were reported according to the ISGPS classification of venous resections.^18^ Aortocaval lymph node resection was defined as harvesting of the lymph node station 16b1, in the aortocaval window. Margin status was classified as microscopically radical resection (>1 mm; R0) and microscopically irradical (≤1 mm; R1).^19^ Lymph node status was reported according to the TNM classification, eighth edition.^20^ Pancreatic surgery–specific complications were all defined according to the ISGPS and the International Study Group of Liver Surgery definitions^3^,^21^–^24^ CL was defined, according to the ISGPS definition, as the output of milky-colored fluid from a drain, drain site, or wound, on or after postoperative day 3, with a triglyceride content of ≥110 mg/dL or ≥1.2 mmol/L^3^. These classifications divide complications in 3 categories (grade A, B, and C) of which only clinically relevant pancreatic surgery complications (grade B/C) were included in the analysis. For CL, grade A has no therapeutic consequences or only oral dietary restrictions; grade B includes nasoenteral nutrition with dietary restriction and/or TPN, percutaneous drainage, maintenance of surgical drains, or drug treatment; grade C other invasive in-hospital treatment, admission to the intensive care unit, and/or mortality.^3^ Postoperative complications were classified according to the Clavien-Dindo classification, of which only complications grade 3 were included. Hospital volume was based on the mean number of PDs performed annually during the study period and was classified into medium (<40 PDs) or high (≥40 PDs). A prolonged length of stay was defined as >14 days, according to the Textbook Outcome definition.^25^

### Statistical Analysis

Baseline characteristics were assessed using descriptive statistics. Results were reported as proportions for categorical variables, and as mean with SD or median with interquartile range (IQR) for continuous variables. Normally distributed data were compared using the Student *t* test, categorical data using the χ^2^ test, and non-normally distributed data using the Mann-Whitney *U* test.

Univariable and multivariable logistic regression models were performed to determine the association between CL and length of stay and in-hospital mortality, adjusted for previously identified risk factors on length of stay, that is, age, American Society Anesthesiologists score, minimally invasive surgery, hospital volume, site of origin, POPF, delayed gastric emptying, postpancreatectomy hemorrhage, pneumonia and wound infection. To identify the potential risk factors for CL using the ISGPS definition, predictors within the patient and pathological characteristics and hospital volume were identified in univariable logistic regression models. Variables with a *P* value <0.20 in univariable analyses were entered in the multivariable regression models and backward step selection was used. Two subgroup analyses were performed to identify risk factors. First, in a patient diagnosed with a malignancy. In this analysis also neoadjuvant therapy, resection margin, and lymph node stadium were taken into account. Second, in patients in whom aortocaval lymph node sampling was registered, to assess whether this was a risk factor CL. Data on para-aortic lymph node resection was only registered in a limited number of patients (participating in a prospective multicenter cohort study about the influence of pancreatic intraoperative nodal status on decision-making during pancreatic surgery) and a limited number of hospitals (10/17). The results are reported as odds ratio (OR) with a corresponding 95% confidence interval (CI). Missing data were reported but not imputed. In multivariable analysis, missing data was excluded.

A *P* value <0.05 was considered statistically significant. Statistical analyses were performed in IBM SPSS Statistics for Windows, version 25 (IBM Corp., Armonk, NY).

## RESULTS

Overall, 2186 patients underwent PD between 2017 and 2019. After the exclusion of 27 patients with missing data on CL, the final cohort consisted of 2159 patients. In this cohort, 46.1% was female and the mean age was 67.1 years (SD=10.4). Of all included patients, 9.8% received neoadjuvant therapy, 20.4% underwent minimally invasive PD, and 16.4% of procedures included a vascular resection (Table 1).

**TABLE 1:** Baseline Characteristics

	All Patients (n=2159)	No CL (n=2007)	CL (n=152)	*P*
Age [mean (SD)] (y)	67.1 (10.4)	67.1 (10.3)	67.4 (11.3)	0.682
Missing	3 (0.1)			
Female	995 (46.1)	926 (46.1)	69 (45.4)	0.859
BMI [mean (SD)]	25.32 (4.4)	25.3 (4.4)	25.2 (4.8)	0.711
Missing	28 (1.3)			
ASA score				
1–2	1511 (70.5)	1406 (70.5)	105 (70.0)	0.887
3–4	632 (29.5)	587 (29.5)	45 (30.0)	
Missing	16 (0.7)			
Neoadjuvant therapy*	176 (8.3)	163 (8.4)	13 (8.9)	0.785
Missing	39 (1.8)			
Preoperative resectability				
Resectable	1734 (85.0)	1308 (83.2)	385 (92.3)	**<0.001**
Borderline resectable	237 (11.6)	201 (12.8)	26 (6.2)	
Locally advanced	70 (3.4)	63 (4.0)	6 (1.4)	
Missing	118 (5.5)			
Minimally invasive PD†	441 (20.9)	429 (21.9)	12 (8.1)	**<0.001**
Missing	53 (2.5)			
PD performed in center with volume ≥40 PD/year‡	1319 (61.1)	1236 (61.6)	83 (54.6)	0.089
Vascular resection				
No	1806 (84.3)	1696 (85.1)	110 (72.8)	**<0.001**
Venous resection ISGPS type 1–2	201 (9.4)	179 (9.0)	22 (14.6)	
Venous resection ISGPS type 3–4	109 (5.1)	92 (4.6)	17 (11.3)	
Arterial resection	21 (1.0)	20 (1.0)	1 (0.7)	
Both arterial and venous resection	6 (0.3)	5 (0.3)	1 (0.7)	
Missing	16 (0.7)			
Additional organ resection§	186 (8.8)	169 (8.6)	17 (11.3)	0.249
Missing	37 (1.9)			

Bold numbers indicates statistical significance (*P*<0.05).

Values are represented as n (%), unless indicated otherwise.

When missing data is not mentioned, there is no missing data.

* In patients with preoperative malignant histology or cytology (n=1751).

† Including patients with conversion to open surgery (n=93).

‡ Volume based on the mean number of PD per year in the study period.

§ Including spleen (intentional or not-intention, mesocolon transversum, colon segment, hemicolectomy, gastric resection, or other).

ASA indicates American Society of Anesthesiologists.

### Incidence and Clinical Impact of CL

Grade B/C CL was present in 152 patients (7.0%), of whom 150 patients (6.9%) were classified as grade B and 2 patients (0.1%) as grade C (Table 2). In other words, the rate of grade C CL among all patients with CL was 2/152 (1.3%).

**TABLE 2:** Postoperative and Pathological Outcomes

	All Patients (n=2159)	No CL (n=2007)	CL (n=152)	*P*
Site of origin				
Pancreas	1193 (56.6)	1110 (56.7)	83 (56.5)	0.761
Distal bile duct	315 (15.0)	292 (14.9)	23 (15.6)	
Ampulla of Vater	339 (16.1)	319 (16.3)	20 (13.6)	
Duodenum or other	259 (12.3)	239 (12.1)	21 (14.3)	
Missing	53 (2.5)			
Postoperative malignant diagnosis	1751 (81.1)	1629 (81.2)	122 (80.3)	0.784
No. lymph nodes [median (IQR)]	15 (11–20)	15 (11–20)	16 (12–22)	0.068
Missing	300 (13.9)			
R1*	588 (33.6)	572 (31.6)	57 (42.2)	**0.011**
Missing	76 (4.3)			
CL, grade B/C	152 (7.0)	NA	NA	NA
POPF, grade B/C	375 (17.4)	351 (17.5)	24 (15.9)	0.611
Missing	5 (0.2)			
Bile leak, grade B/C	129 (6.1)	122 (6.2)	7 (4.7)	0.461
Missing	27 (1.3)			
Delayed gastric emptying, grade B/C	442 (20.7)	412 (20.8)	30 (19.7)	0.763
Missing	23 (1.1)			
Postpancreatectomy hemorrhage, grade B/C	172 (8.1)	157 (7.9)	15 (10.0)	0.367
Missing	27 (1.3)			
Postoperative complications Clavien-Dindo ≥3	688 (31.9)	NA	NA	NA
Missing	39 (1.8)			
Pneumonia	132 (6.2)	123 (6.2)	9 (6.2)	0.998
Missing	31 (1.4)			
Wound infection	243 (11.5)	220 (11.2)	23 (15.5)	0.108
Missing	42 (1.9)			
Length of hospital stay [median (IQR)]	12 (8–19)	12 (8–19)	16 (11–25)	**<0.001**
Missing	11 (0.5)			
Mortality	58 (2.7)	55 (2.7)	3 (2.0)	0.572
Missing	2 (0.1)			

Values are represented as n (%), unless indicated otherwise.

When missing data is not mentioned, there is no missing data.

*In patients with preoperative malignant histology or cytology (n=1751).

NA indicates not applicable.

Bold numbers indicates statistical significance (*P*<0.05).

A postoperative complication (Clavien-Dindo ≥3) occurred in 688 patients (31.9%). The pancreatic-specific grade B/C complications, occurred in 375 patients for POPF (17.4%), 129 bile leak (6.0%), 442 delayed gastric emptying (20.5%), and 172 postpancreatectomy hemorrhage (8.0%). CL occurred in combination with other postoperative complications in 48.6%, mainly in combination with delayed gastric emptying (19.7%) and POPF (15.9%). Patients with CL had a longer median length of stay compared with patients without CL [16.0 days (IQR: 11–25) vs 12.0 days (IQR: 8–19), respectively, *P*<0.001]. Mortality did not differ significantly between patients with and without CL [n=3/152 (2.0%) vs n=55/2007 (2.7%), *P*=0.572]. In multivariable logistic regression, CL was independently associated with a prolonged length of stay (OR=2.8, 95% CI: 1.9–4.4, *P*<0.001, Table 3). Moreover, CL was not associated with in-hospital mortality (OR=0.3, 95% CI: 0.04–2.3, *P*=0.244, Table 4).

**TABLE 3:** Multivariable Logistic Regression to Assess Association With Prolonged Length of Stay (>14 Days) in Patients After PD

	Multivariable Analysis* [OR (95% CI)]	*P*
Age ≥70	1.05 (0.82–1.33)	0.710
ASA ≥3	1.51 (1.16–1.96)	0.002
Minimally invasive surgery	0.86 (0.63–1.17)	0.330
PD performed in center with volume ≥40 PD/year†	0.71 (0.56–0.91)	0.007
Site of origin		
Pancreas	Reference	
Distal bile duct	1.21 (0.86–1.70)	0.277
Ampulla of Vater	1.08 (0.77–1.52)	0.666
Duodenum or other	1.79 (1.25–2.58)	0.002
CL grade B/C	2.84 (1.85–4.36)	**<0.001**
POPF grade B/C	7.37 (5.21–10.42)	<0.001
Bile leak grade B/C	12.15 (6.53–22.61)	<0.001
Delayed gastric emptying grade B/C	11.76 (8.59–16.10)	<0.001
Postpancreatic hemorrhage grade B/C	2.36 (1.42–3.90)	0.001
Pneumonia	1.86 (1.09–3.18)	0.023
Wound infection	2.15 (1.49–3.10)	0.007

Bold numers indicate the relationship of CL with the outcome investigated.

* Multivariable logistic analysis in 1907 patients.

† Volume based on the mean number of PD per year in the study period.

ASA indicates American Society of Anesthesiologists.

**TABLE 4:** Multivariable Logistic Regression to Assess the Association With CL and In-hospital Mortality in Patients After PD

	Multivariable Analysis* [OR (95% CI)]	*P*
Age ≥70	1.95 (0.99–3.81)	0.051
ASA ≥3	2.06 (1.08–3.92)	0.028
Minimally invasive surgery	0.78 (0.34–1.78)	0.553
PD performed in center with volume ≥40 PD/year†	0.45 (0.23–0.88)	**0.020**
Site of tumor		
Pancreas	Reference	
Distal bile duct	0.36 (0.11–1.39)	0.149
Ampulla of Vater	0.69 (0.27–1.81)	0.460
Duodenum or other	1.70 (0.74–3.91)	0.211
CL grade B/C	**0.30 (0.04**–**2.27)**	**0.244**
POPF grade B/C	1.28 (0.54–3.01)	0.578
Bile leak grade B/C	2.57 (1.02–6.48)	0.045
Delayed gastric emptying grade B/C	0.87 (0.39–1.92)	0.727
Postpancreatic hemorrhage grade B/C	6.42 (3.02–13.72)	<0.001
Pneumonia	2.00 (0.77–5.23)	0.157
Wound infection	0.46 (0.13–1.59)	0.222

Bold numers indicate the relationship of CL with the outcome investigated.

* Multivariable logistic analysis in 1916 patients.

† Volume based on the mean number of PD per year in the study period.

ASA indicates American Society of Anesthesiologists.

### Predictors for CL

The univariable analysis identified open surgery, vascular resection, additional resection, hospital volume, tumor diameter, and >15 lymph nodes resected as predictors for CL (Table 5). After multivariable analysis, only vascular resection (OR=2.1, 95% CI: 1.4–3.2, *P*<0.001) and open surgery (OR=3.5, 95% CI: 1.7–7.2, *P*=0.001) were independent predictors for CL. When using subgroups for the type of vascular resection, both ISGPS type 1 to 2 vascular resection and type 3 to 4 vascular resection were identified as risk factors for CL, whereas an arterial vascular resection was not (Supplementary Table 1, Supplemental Digital Content 1, http://links.lww.com/SLA/D946). There were significant (baseline) differences between patients after open and minimally invasive surgery, namely in terms of preoperative resectability, hospital volume, vascular resections, and additional organ resections (Supplementary Table 2, Supplemental Digital Content 2, http://links.lww.com/SLA/D947).

**TABLE 5:** Multivariable Regression Analysis to Assess Predictors for CL in PD

	Univariable Analysis [OR (95% CI)]	*P*	Multivariable Analysis* [OR (95% CI)]	*P*
Age ≥70	1.13 (0.82–1.58)	0.454		
Female	0.97 (0.69–1.35)	0.859		
ASA ≥3	1.23 (0.72–1.48)	0.887		
BMI	0.99 (0.96–1.03)	0.711		
Preoperative resectability†				
Resectable	Reference			
Borderline resectable	1.19 (0.72–1.98)	0.492		
Locally advanced	1.28 (0.55–3.03)	0.568		
Open surgery	**3.18 (1.75**–**5.79)**	**<0.001**	**3.49 (1.68**–**7.24)**	**0.001**
Vascular resection	**2.14 (1.46**–**3.12)**	**<0.001**	**2.12 (1.39**–**3.21)**	**<0.001**
Additional resection	1.36 (0.80–2.31)	0.250		
PD performed in center with volume ≥40 PD/year‡	**0.75 (0.54**–**1.05)**	**0.090**		
Site of origin				
Pancreas	Reference			
Distal bile duct	1.05 (0.65–1.70)	0.832		
Ampulla of Vater	0.84 (0.51–1.39)	0.493		
Duodenum or other	1.18 (0.72–1.94)	0.515		
Malignant diagnosis	1.06 (0.70–1.60)	0.784		
≥15 lymph nodes resected†	**0.75 (0.52**–**1.09)**	**0.133**		
POPF grade B/C	0.89 (0.57–1.39)	0.611		

Bold numbers in univariable analysis indicates variables that were entered in multivariable analysis (*P*<0.20). Bold numbers in multivariable analysis indicates statistical significance (*P*<0.05).

* Multivariable analysis after backward step selection in 1809 patients.

† Value used is the median number of lymph nodes resected.

‡ Volume based on the mean number of PD per year in the study period.

ASA indicates American Society of Anesthesiologists.

In the subgroup of patients with malignancy, the additionally assessed risk factors (ie, neoadjuvant therapy, resection margin, and lymph node stadium) were not associated with CL. Only vascular resection (OR=1.8, 95% CI: 1.2–2.8, *P*=0.016) and open surgery (OR=3.7, 95% CI: 1.6–8.5, *P*=0.002) were independent risk factors for CL (Supplementary Table 3, Supplemental Digital Content 3, http://links.lww.com/SLA/D948). In a subgroup analysis of patients for which aortocaval lymph node sampling was registered (n=456), aortocaval lymph node sampling was not identified as a risk factor for CL in multivariable analysis (Supplementary Table 3, Supplemental Digital Content 3, http://links.lww.com/SLA/D948).

## DISCUSSION

This first nationwide study demonstrated a 7.0% incidence of ISGPS grade B/C CL, with CL grade C being extremely rare (0.1%; 1.3% of all CL). CL was associated with prolonged length of hospital stay but not with mortality. Vascular resection and open surgery were independent risk factors for CL.

The 7.0% rate of CL in the present study falls within the range of the previous studies in which it ranges from 0.6% to 16.3%.^4^–^9^ This wide range in previous studies, illustrates the relevance of a uniform classification and definition of CL to allow accurate comparison of outcomes across institutions and countries. The results of the present study were comparable to a previous monocenter validation study of the ISGPS CL definition.^11^ Also in that study, grade C CL was extremely rare (0.2% vs 0.1% in the present study) with grade B CL being much more common (3.3% vs 6.9% in the present study). The rate of CL was slightly higher in our study than in the monocenter study, which could be explained by the higher number of vascular resections in this study (12.7% vs 16.1% in the present study) possibly caused by the different time periods (2014–2016 vs 2017–2019), or by the early enteral feeding protocol via the enhanced recovery after surgery model which is incorporated in the Dutch guidelines and clinical practice.^26^,^27^ Both studies also confirmed the relation between CL and prolonged length of hospital stay. Moreover, the monocenter study did not identify intraoperative factors associated with CL. This study was, however, not powered to identify risk factors.

Given the very low rate of ISGPS grade C CL, the main question is whether the ISGPS classification of CL should be changed. The fact that grade C CL is extremely rare in both validation studies, suggests that this part of the classification is not of additional value, but also confirms that CL mostly runs a relatively mild clinical course and rarely becomes life-threatening. Thus, it could be debated to redefine grade C. For example, percutaneous drainage and TPN could be classified as grade C, as this can be considered more invasive treatment than maintenance of surgical drains or nasoenteral nutrition with dietary restrictions. However, when assessing this point in relation to the other ISGPS definitions, such as delayed gastric emptying, TPN should remain as a grade B complication.^21^ Furthermore, also in most other ISGPS classifications, grade A complications have no to very limited clinical consequences and grade C complications reflect a life-threatening condition. The latter is clearly not the case in patients with CL and so justifies the current type C definition. One could debate whether to leave out grade A altogether such as was done in the ISGPS definition of pancreatic fistula.^23^ Our group supports the notice that the low rate of grade C CL is actually reassuring and this definition could remain intact to stay in line with other ISGPS definitions.

Vascular resection and open surgery were identified as risk factors for CL in the present study. Vascular resection is a commonly identified predictor and can easily explain the higher rate of CL.^6^–^8^ In an extensive operation, there is more risk on damage to the main abdominal lymphatic vessels.^2^ Only venous resections are identified as significant risk factors and arterial resection are not, although there only was a limited number of arterial resections performed in this cohort, shown by the broad CI (Supplementary Table 1, Supplemental Digital Content 1, http://links.lww.com/SLA/D946). Our study is the first to report on open surgery as being a risk factor for CL. However, this could very will still be due to patient selection. The literature is conflicting on this point. A single-center retrospective study specifically investigating CL in open (n=118) versus robot PD (n=165), found no effect of the surgical approach on CL (10.9% vs 13.6%, *P*=0.449).^28^ In the 4 randomized trials on laparoscopic versus open PD, only the multicenter randomized LEOPARD-2 trial reported on the incidence of CL.^29^–^32^ A nonsignificant increase in CL after laparoscopic as compared with open PD (n=2/50 [4%] vs n=7/49 [14%], *P*=0.09) was reported.^30^ In general, minimally invasive PD will be reserved for less advanced tumors without vascular contact. For example, in the period 2016 to 2019 the LEALAPS-3 multicenter training program for robot PD was performed in The Netherlands, in which BMI ≤35 kg/m^2^ and no vascular involvement were eligibility criteria.^33^ This is also confirmed by our analysis that shows a significant difference in preoperative resectability, vascular resection, and additional organ resections between the minimally invasive and open surgery group (Supplementary Table 2, Supplemental Digital Content 2, http://links.lww.com/SLA/D947). Thereby, in patients with malignancy, the rate of CL was nonsignificantly increased in patients with an R1 resection (OR=1.44, 95% CI: 0.97–2.15, *P*=0.70), especially in patients in whom aortocaval lymph node sampling took place (OR=3.43, 95% CI: 1.65–7.12, *P*=0.001). The latter is actually an important finding and should be taken into account when sampling aortocaval lymph nodes. The association between CL and R1 resection can possibly be explained by a more extensive resection being performed in these patients. A recent review and meta-analysis shows that R1 resection was associated with advanced tumor disease, namely larger tumor size, lymph node metastases, and extended resections.^34^

Some of the previously identified risk factors were not confirmed by our analysis, that is, age, sex, lymph node status, number of resected lymph nodes, aortocaval lymph node sampling, and concomitant POPF.^7^–^9^,^11^,^13^,^35^ This could partly be explained by the different (older) definitions of CL used in the previous studies. It is of specific interest that the number of resected lymph nodes and aortocaval lymph node sampling were not found as a risk factor in our analysis, since this would directly cause damage of lymphatic vessels and cisterna chyli. This could be due to the fact that extended lymph node dissection in patients with (borderline) resectable pancreatic cancer is infrequently used.^27^ However, this cannot be put with certainty, as there is no distinction between regular and extended lymph node dissection in this analysis.

Unfortunately, the identified risk factors are difficult to prevent, because limiting the extent of resection could result in nonradical resection.^36^ In the case of portovenous vascular involvement, a vascular resection is required, thus accepting the higher risk of CL. Therefore, specific focus on the treatment of CL should be considered. However, data on management of CL are limited, as is also the case in the present study, and show no consensus. The ISGPS definition paper suggests a step-up approach, starting with dietary restrictions (a diet restricted in long-chain triglycerides, or a no-fat diet with MCT supplementation); if this does not lead to decreased drain output, TPN may be considered; if TPN treatment fails, more invasive treatment options should be considered such as: sclerotic embolization, a peritoneovenous shunt or the use of lymphangiography for operative ligation.^3^ Another study describes a conservative treatment with a combination of customized enteral feeds, supplemental parenteral nutrition, and octreotide as a successful treatment. Of all 159 patients evaluated only one patient required additional percutaneous drainage, the rest could be managed conservatively.^37^ One prospective study investigated different treatment options (no treatment, MCT diet, TPN, and a combination of MCT diet and TPN) in 228 patients, in which type of treatment had no effect on time to drain removal and morbidity. Furthermore, morbidity was not increased in patients who had their drains removed despite persistent CL (*P*=0.84). However, when interpreting these results, it should be taken into account that decision-making about drain removal remained at the surgeon’s discretion in this study.^12^ In conclusion, there is no consensus yet about the treatment of CL.

This study has several limitations. First, as this is a retrospective study there is a risk of selection bias, for instance, data on aortocaval lymph node sampling was only registered in a limited number of patients. Second, data on the management of CL (eg, nasoenteral nutrition, total parenteral nutrition, octreotide, or percutaneous drainage), on nutrition (eg, early enteral feeding), variables predicting an a course that requires an aggressive approach (eg, lymphopenia), and some potential predictors in previous studies (eg, portal/mesenteric thrombosis and operative time) are not registered in the DPCA and therefore not available for this analysis.^4^,^6^,^7^,^10^ Third, the intended extent of lymph node dissection is not available in the audit. Therefore, no distinction can be made between regular lymph an extended resection as a risk factor for CL. Strengths of this study include the large size, nationwide aspect, and the dedicated registration of CL according to the ISGPS definition within the registry.

In conclusion, this nationwide post hoc assessment of the prospectively maintained nationwide audit shows that the rate of CL according to the ISGPS B/C definition is 7.0% with grade C being extremely rare (0.1%). The use of this definition is recommended for further studies, to adequately compare results. Risk factors for CL should be considered in the postoperative follow-up period. Moreover, a future step would be to study strategies to prevent CL and, mostly, to achieve consensus about the treatment.

## Supplementary Material
