# Supplementary material for: Chyle Leak After Pancreatoduodenectomy: Clinical Impact and Risk Factors in a Nationwide Analysis
Source: Ann Surg. 2022 Jul 4;277(6):e1299–305. doi: 10.1097/SLA.0000000000005449 (PMC10174101; doi:10.1097/SLA.0000000000005449)
Supplement: Supplementary file 1 [file sla-277-e1299-s001.docx]

**SUPPLEMENTARY TABLE 1**. Multivariable regression analysis to assess predictors for chyle leak in patients after pancreatoduodenectomy including details on vascular resection

|  | **Univariable analysis  OR (95%CI)** | **P-value** | **Multivariable analysis^a^ OR (95% CI)** | **P-value** |
| --- | --- | --- | --- | --- |
| Age ≥70 | 1.13 (0.82-1.58) | 0.454 |  |  |
| Female | 0.97 (0.69-1.35) | 0.859 |  |  |
| ASA ≥3 | 1.23 (0.72-1.48) | 0.887 |  |  |
| BMI | 0.99 (0.96-1.03) | 0.711 |  |  |
| Pre-operative resectability  Resectable  Borderline resectable  Locally advanced | reference 1.19 (0.72-1.98) 1.28 (0.55-3.03) | 0.492 0.568 |  |  |
| Open surgery | **3.18 (1.75-5.79)** | **<0.001** | **3.49 (1.68-7.26)** | **0.001** |
| Vascular resection  No vascular resection  Venous wedge ISGPS type 1-2  Venous segment ISPS type 3-4  Arterial resection  Both arterial and venous | **Reference 1.89 (1.17-3.07) 2.85 (1.64-4.95)** 0.77 (0.10-5.79) 3.08 (0.36-26.62) | **0.009 <0.001** 0.800 0.306 | **Reference 1.70 (1.01-2.88) 2.82 (1.58-5.04)** 0.92 (1.12-7.01) 4.66 (0.48-45.29) | **0.048 <0.001** 0.935 0.185 |
| Additional resection | 1.36 (0.80-2.31) | 0.250 |  |  |
| PD performed in center with volume ≥40 PD/year^b^ | **0.75 (0.54-1.05)** | **0.090** |  |  |
| Site of origin  Pancreas  Distal bile duct  Ampulla of Vater  Duodenum or other | reference 1.05 (0.65-1.70) 0.84 (0.51-1.39) 1.18 (0.72-1.94) | 0.832 0.493 0.515 |  |  |
| Malignant diagnosis | 1.06 (0.70-1.60) | 0.784 |  |  |
| ≥15 lymph nodes resected^c^ | **0.75 (0.52-1.09)** | **0.133** |  |  |
| Post-operative pancreatic fistula grade B/C | 0.89 (0.57-1.39) | 0.611 |  |  |

Bold numbers in univariable analysis indicates variables that were entered in multivariable analysis (p<0.20). Bold numbers in multivariable analysis indicates statistical significance (p<0.05). OR: Odds ratio; ASA: American Society of Anesthesiologists. ^a^Multivariable analysis after backward step selection in 1807 patients. ^b^Volume based on the mean number of pancreatoduodenectomy per year in the study period. ^c^Value used is the median number of lymph nodes resected.
