# Supplementary material for: Chyle Leak After Pancreatoduodenectomy: Clinical Impact and Risk Factors in a Nationwide Analysis
Source: Ann Surg. 2022 Jul 4;277(6):e1299–305. doi: 10.1097/SLA.0000000000005449 (PMC10174101; doi:10.1097/SLA.0000000000005449)
Supplement: Supplementary file 2 [file sla-277-e1299-s002.docx]

**SUPPLEMANTARY TABLE 2.** Baseline characteristics minimally invasive surgery

|  | **Open  (n= 2106)** | **MIS  (n=441)** | **P-value** |
| --- | --- | --- | --- |
| Age, mean years (SD) | 66.9 (10.4) | 67.8 (10.2) | 0.113 |
| Female | 768 (46.1%) | 203 (46.0%) | 0.972 |
| BMI, mean (SD) | 25.3 (4.5) | 25.4 (3.9) | 0.814 |
| ASA score   1-2  3-4 | 1038 (67.8%) 492 (32.2%) | 277 (68.6%) 127 (31.4%) | 0.782 |
| Neoadjuvant therapy^a^ | 142 (16.0%) | 25 (11.2%) | 0.071 |
| Pre-operative resectability   Resectable  Borderline resectable  Locally advanced | 1308 (83.2%) 201 (12.8%) 63 (4.0%) | 385 (92.3%) 26 (6.2%)  6 (1.4%) | **<0.001** |
| PD performed in center with volume ≥40 PD/year^b^ | 992 (59.6%) | 296 (67.1%) | **0.004** |
| Vascular resection   No  Venous resection ISGPS type 1-2  Venous resection ISGPS type 3-4  Arterial resection  Both arterial and venous resection | 1360 (82.2%) 176 (10.6%) 96 (5.8%) 16 (1.0%)  6 (0.4%) | 407 (92.3%) 19 (4.3%)  11 (2.5%) 4 (0.9%)  0 (0.0%0 | **<0.001** |
| Additional organ resection^c^ | 164 (10.0%) | 17 (3.9%) | **<0.001** |

Values are numbers with valid percentages unless indicated otherwise. Bold numbers indicate statistical significance (p<0.05). ASA: American Society of Anesthesiologist, BMI: body mass index, SD: standard deviation, MIS: minimally invasive surgery. ^a^In patients with pre-operative malignant histology or cytology. ^b^Volume based on the mean number of pancreatoduodenectomy per year in the study period. ^c^Including spleen (intentional or not-intention, mesocolon transversum, colon segment, hemi-colectomy, gastric resection or other)
