# Supplementary material for: Chyle Leak After Pancreatoduodenectomy: Clinical Impact and Risk Factors in a Nationwide Analysis
Source: Ann Surg. 2022 Jul 4;277(6):e1299–305. doi: 10.1097/SLA.0000000000005449 (PMC10174101; doi:10.1097/SLA.0000000000005449)
Supplement: Supplementary file 3 [file sla-277-e1299-s003.docx]

***SUPPLEMENTARY TABLE 3****. Multivariable regression analysis to assess predictors for chyle leak in pancreatoduodenectomy for patients with malignant disease*

|  | **Patients with malignant disease** | | | | **Patients with aortocaval lymph node sampling** | | | |
| --- | --- | --- | --- | --- | --- | --- | --- | --- |
|  | **Univariable analysis  OR (95%CI)** | **P-value** | **Multivariable analysis^a^ OR (95% CI)** | **P-value** | **Univariable analysis  OR (95%CI)** | **P-value** | **Multivariable analysis^b^ OR (95% CI)** | **P-value** |
| Age ≥70 | 1.14 (0.79-1.64) | 0.495 |  |  | 0.69 (0.37-1.30) | 0.254 |  |  |
| Female | 0.85 (0.59-1.23) | 0.394 |  |  | 0.95 (0.52-1.77) | 0.880 |  |  |
| ASA ≥3 | 1.04 (0.70-1.55) | 0.830 |  |  | 0.66 (0.29-1.46) | 0.306 |  |  |
| BMI | 0.99 (0.94-1.03) | 0.525 |  |  | 1.02 (0.95-1.09) | 0.610 |  |  |
| Pre-operative resectability  Resectable  Borderline resectable  Locally advanced | reference 1.28 (0.76-2.17) 1.36 (0.57-3.23) | 0.355 0.487 |  |  | reference 0.78 (0.29-2.06) 0.68 (0.09-5.30) | 0.618 0.716 |  |  |
| Neoadjuvant chemo(radio)therapy | 1.16 (0.64-2.11) | 0.633 |  |  | 0.82 (0.28-2.39) | 0.721 |  |  |
| Open surgery | **3.69 (1.78-7.64)** | **0.000** | **3.72 (1.70-8.09)** | **0.001** | NA^c^ |  |  |  |
| Vascular resection | **2.10 (1.39-3.16)** | **0.000** | **1.67 (0.97-2.14)** | **0.021** | 0.94 (0.43-2.09) | 0.888 |  |  |
| Additional resection | **1.58 (0.91-2.74)** | **0.107** |  |  | 0.86 (0.25-2.90) | 0.807 |  |  |
| PD performed in center with volume ≥40 PD/year^d^ | **0.75 (0.52-1.09)** | **0.133** |  |  | **0.29 (0.16-0.56)** | **<0.001** | **5.33 (2.56-11.08)** | **<0.001** |
| Aortocaval lymph node sampling | NA | NA |  |  | **2.73 (1.37-5.44)** | **0.004** |  |  |
| Site of origin  Pancreas  Distal bile duct  Ampulla of Vater  Duodenum or other | reference 1.01 (0.61-1.67) 0.71 (0.39-1.26)  1.08 (0.59-2.00) | 0.971  0.237  0.799 |  |  | reference 1.14 (0.47-2.75) 1.26 (0.58-2.73) 0.89 (0.26-3.09) | 0.771 0.560 0.859 |  |  |
| Pancreatic ductal adenocarcinoma | 1.17 (0.80-1.69) | 0.419 |  |  | 0.81 (0.24-2.73) | 0.734 |  |  |
| R1 resection | **1.74 (1.19-2.54)** | **0.004** | 1.44 (0.97-2.15) | 0.70 | **2.09 (1.12-3.91)** | **0.020** | **3.43 (1.65-7.13)** | **0.001** |
| N stadium  N0  N1  N2 | reference 1.09 (0.69-1.72) 1.27 (0.80-2.01) | 0.697 0.307 |  |  | reference 1.12 (0.49-2.55) 1.83 (0.83-4.03) | 0.796 0.137 |  |  |
| Lymph nodes resected >15^e^ | 1.26 (0.87-1.82) | 0.224 |  |  | **2.21 (1.12-4.37)** | **0.023** |  |  |
| Post-operative pancreatic fistula grade B/C | 0.76 (0.44-1.30) | 0.318 |  |  | **0.34 (0.10-1.12)** | **0.075** |  |  |

Bold numbers in univariable analysis indicates variables that were entered in multivariable analysis (p<0.20). Bold numbers in multivariable analysis indicates statistical significance (p<0.05). OR: Odds ratio; ASA: American Society of Anesthesiologists, NA: not applicable. ^a^Multivariable analysis after backward step selection in 1751 patients. ^b^Multivariable analysis after backward step selection in 405 patients. ^c^Open surgery could not be included in this analysis, due to the fact that only 2 patients with CL underwent minimally invasive surgery. ^d^Volume based on the mean number of pancreatoduodenectomy per year in the study period. ^e^Value used is the median number of lymph nodes resected.
